# Downfalls of Chemical Probes Acting at the Kinase ATP-Site: CK2 as a Case Study

**DOI:** 10.3390/molecules26071977

**Published:** 2021-03-31

**Authors:** Eleanor L. Atkinson, Jessica Iegre, Paul D. Brear, Elizabeth A. Zhabina, Marko Hyvönen, David R. Spring

**Affiliations:** 1 Department of Chemistry, University of Cambridge, Lensfield Road, Cambridge CB2 1EW, UK; ela32@cam.ac.uk (E.L.A.); ji243@cam.ac.uk (J.I.); 2 Department of Biochemistry, University of Cambridge, 80 Tennis Court Road, Cambridge CB2 1GA, UK; pdb47@cam.ac.uk (P.D.B.); eaz20@cam.ac.uk (E.A.Z.); mh256@cam.ac.uk (M.H.)

**Keywords:** kinase, CK2, inhibitor, cancer, molecular probe, ATP-site, COVID-19

## Abstract

Protein kinases are a large class of enzymes with numerous biological roles and many have been implicated in a vast array of diseases, including cancer and the novel coronavirus infection COVID-19. Thus, the development of chemical probes to selectively target each kinase is of great interest. Inhibition of protein kinases with ATP-competitive inhibitors has historically been the most widely used method. However, due to the highly conserved structures of ATP-sites, the identification of truly selective chemical probes is challenging. In this review, we use the Ser/Thr kinase CK2 as an example to highlight the historical challenges in effective and selective chemical probe development, alongside recent advances in the field and alternative strategies aiming to overcome these problems. The methods utilised for CK2 can be applied to an array of protein kinases to aid in the discovery of chemical probes to further understand each kinase’s biology, with wide-reaching implications for drug development.

## 1. Introduction

Protein kinases are a large class of enzymes which add a phosphate group from ATP to serine, threonine or tyrosine residues in other protein substrates [1]. The vast majority of cellular signalling pathways and processes are regulated by the phosphorylation of proteins carried out by protein kinases [2,3]. As such, it is unsurprising that protein kinases have become key targets for pharmaceutical development for a wide range of diseases, the most extensively studied being cancer [1,4,5]. According to the protein kinase inhibitor database (PKIDB), as of January 2021 there were 63 FDA-approved drugs targeting protein kinases, in addition to a further 186 compounds in clinical trials; 8 of the drugs were approved by the FDA in 2020, highlighting the rapidly evolving nature of the field [6,7].

It is estimated that 30–50% of proteins may undergo phosphorylation [2,8]. Phosphorylation by protein kinases has a multitude of effects including, but not limited to, regulation of enzyme activity. The human genome contains 518 genes that encode for serine/threonine/tyrosine kinases, each with specific substrate specificity, activity and stability; as one of the largest protein families, protein kinases make up around 2% of the entire human protein-encoding genome [9,10,11]. Protein kinases can be identified by their conserved catalytic domains, with the structure of the ATP binding site in particular showing great similarity between all kinases [12].

### 1.1. Structure of the ATP Binding Site of Protein Kinases

**Figure 1:**
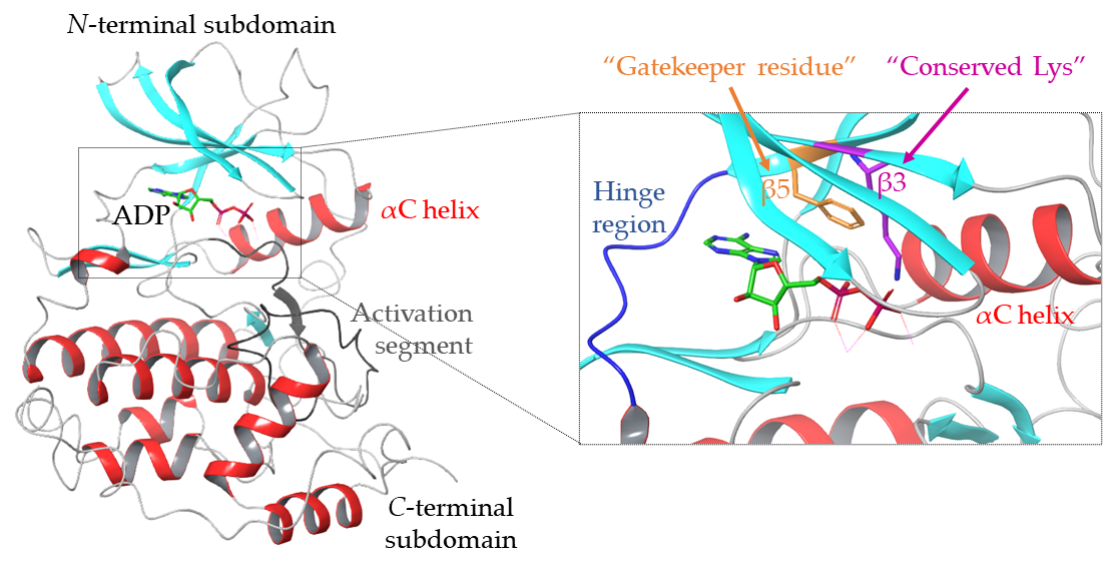
Crystal structure of the protein kinase CK2 α-subunit with ADP bound in the catalytic site (PDB: 6YPN). α-helices are in red; β-strands are in cyan; the activation segment is in dark grey; the hinge region is in blue; the conserved Lys in the β3-strand (Lys68) is in magenta; the gatekeeper residue in the β5-strand (Phe113) is in orange.

The catalytic domain of protein kinases consists of two parts, the *N*-and *C*-terminal subdomains; it is between these two subdomains, which are connected by a hinged linker, that the ATP-site is formed. The *N*-terminal subdomain is mainly comprised of β-strands which close off the top of the active site, as well as a crucial α-helix, (αC); the *C*-terminal subdomain predominantly consists of α-helices (Figure 1). A “gatekeeper” residue in β-strand 5 (β5) and a Lys in β3 form a divider, or a “gate”, between the front and the back of the catalytic pocket (Figure 1). Access to the back of the pocket is controlled by the gatekeeper residue. If the gatekeeper is small (e.g., Ala) the back of the pocket is accessible; however, if it is a bulky residue (e.g., Phe), entry to the back of the catalytic pocket is obstructed. The Lys residue in the β3 strand (Figure 1) is conserved in all kinases and is necessary for the catalytic activity of the enzymes; the Lys helps to secure the phosphates of ATP in the active site [13,14,15,16,17]. The hinged linker contains a highly conserved Gly rich motif (GXGXδG where δ is usually Tyr or Phe) (Figure 1). The small size of the Gly residues allows the linker to come into close proximity with ATP and favourable interactions are formed between the peptide backbone and the phosphate groups of ATP [15,16,17].

### 1.2. Problems with ATP-Competitive Inhibitors

Due to the well understood structure of the ATP sites of numerous protein kinases, as well as the obvious effect on catalytic activity of the site, the development of ATP-competitive protein kinase inhibitors has been an active field over the past few decades. However, due to the highly conserved nature of the ATP-site, selective inhibition of protein kinases via active site inhibitors is extremely challenging to achieve [18,19]. A large number of inhibitors targeting the ATP-site bind with donor-acceptor interactions to the conserved hinge region of the protein that naturally binds the adenine moiety of ATP; binding to this region can produce strong interactions but often leads to more indiscriminate inhibitors [20].

One of the first ATP-competitive inhibitors identified was staurosporine, a microbial alkaloid which in 1986 was found to inhibit protein kinase C (PKC, IC_50_ = 2.7 nM) [21]. At the turn of the century, many cell permeable protein kinase inhibitors were regarded as “selective” and were being used in cell assays to investigate the biological roles of the protein kinases they target. However, in 2000 Davies et al. investigated the selectivity of 28 commercially available “selective” protein kinase inhibitors and found that, excluding rapamycin and PD 184352, all other inhibitors affected at least one other protein kinase, with most altering the activity of multiple. They also highlighted that it is frequently not just closely related kinases in the same family that are affected [22]. These results were further built upon by Karaman et al. who screened 38 kinase inhibitors against 317 kinases. Astonishingly, they identified staurosporine as a strong inhibitor of over 90% of the kinase panel tested and thus, it was deemed to be a pan-kinome inhibitor [18]. Although this is an extreme example, it highlights the great similarity amongst the kinome and the promiscuous nature of many ATP-competitive inhibitors.

An alternative method of ATP-site inhibition developed by the Shokat group involves mutating the highly conserved gatekeeper residue of a protein kinase to a small Gly or Ala to create a uniquely targetable mutant kinase [23,24]. This in theory facilitates the identification of specific inhibitors for each kinase. However, the inhibitors are typically only effective against the mutant kinases and thus, although an effective method for specifically modulating the activity of individual kinases, it does not represent a valid therapeutic strategy at this time.

Despite the challenges in their development, successful ATP-competitive protein kinase inhibitors have been developed, with more recently being approved for clinical use by the FDA [25,26,27,28,29]. However, the majority of these compounds have undesirable side effects and are used for the second-line treatment of cancers, where initial treatments have been unsuccessful [25,26,27,28,29,30,31]. These compounds are of great clinical importance; however, the ultimate goal is the development of fully selective inhibitors with minimal off-target effects, making for a highly targeted therapeutic which is applicable in more than just the worst-case scenarios. Additionally, effective chemical probes for a wide variety of proteins are required to thoroughly investigate biological pathways; compounds deemed sufficiently selective as therapeutic agents may still not be effective chemical probes, providing convoluted results arising from the modulation of the activity of more than one protein. As such, highly selective molecules are needed to successfully determine the specific roles that different proteins play in the biochemistry of living organisms.

### 1.3. Protein Kinase CK2

Casein kinase 2, now commonly known as CK2, is a ubiquitous serine/threonine kinase belonging to the CMGC group of kinases [32,33]. CMGC kinases are typically involved in cell-cycle regulation, cell signalling, cell communication and cell growth [34]. CK2 phosphorylates over 300 substrates and, as with other CMGC kinases, has multiple roles in the cell cycle, including in cell growth, proliferation and survival [35,36,37]. Interestingly, unlike most other protein kinases, CK2 is constitutively active; it does not require phosphorylation to exert its function [38]. One of CK2′s key roles in the cell is as an anti-apoptotic protein and, as such, it is seen to be overexpressed in a wide variety of cancerous tumours including, but not limited to, lung, colorectal and prostate cancers [39,40,41,42]. Both healthy and cancer cells make use of CK2, however, cancer cells are particularly sensitive to CK2 inhibition due to the absence of alternative pathways to compensate for its downregulation [38,43,44]. Promisingly, a first-in-class small molecule inhibitor, CX-4945, is currently in phase II clinical trials for the treatment of cholangiocarcinoma [45]. It should also be noted that, although the role of CK2 in cancer has been the most widely studied, CK2 is also implicated in many other diseases, including COVID-19 [46,47].

**Figure 2:**
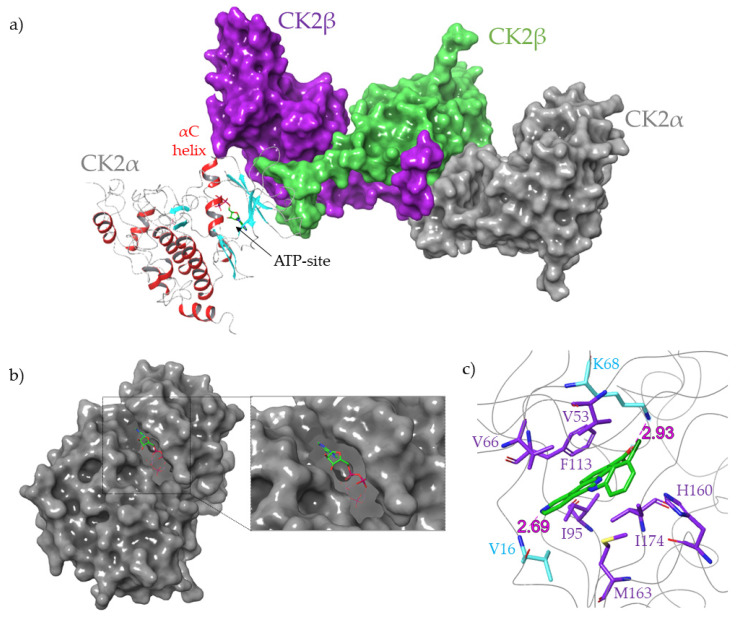
Overview of the structure of the CK2 holoenzyme, the catalytic subunit CK2α with ADP bound in the ATP-site and the key interactions between residues in the ATP-site of CK2α and the ATP-competitive inhibitor CX-4945. (**a**) Heterotetrameric quaternary structure of CK2 with AMPPNP bound in the ATP-site of CK2α (PDB: 1JWH). The catalytic α subunits are shown in grey and in ribbon form; α-helices are shown in red and β-strands are shown in cyan in the ribbon form; the regulatory β subunits are shown in purple and green. (**b**) Structure of CK2α with ADP bound in the ATP-site (PDB: 6YPN). (**c**) Structure of ATP-competitive inhibitor CX-4945 bound in the ATP-site of CK2α (PDB: 3NGA). CX-4945 is shown in green. Residues which form hydrogen bonds with the inhibitor are shown in cyan with the distances between the residues and the inhibitor shown in Å; residues which form van der Waals interactions with the inhibitor are shown in purple.

CK2 has a heterotetrameric quaternary structure made up of two catalytic kinase subunits (α and/ or α’) and two regulatory subunits (β) (Figure 2) [42,43,48]. CK2α and CK2α’ are coded for by distinct genes and differ in their sequences mostly at the *C*-terminus (CK2α has a longer *C*-terminal extension). They both act as catalytic units in their own right and can come together in any combination to form the heterotetrameric holoenzyme, i.e.*,* ααββ, α’αββ, α’α’ββ [49]. The β subunits control the substrate specificity and cellular localisation of the enzyme, as well as providing stability, while the α subunits phosphorylate the enzyme’s substrates [50]. The structure of the catalytic subunit resembles that of a generic protein kinase, as described previously (Figure 2).

CK2α exists only in an active conformation without the need for upstream phosphorylation, unlike the majority of protein kinases which have an “on” and “off” form, reflecting its constitutive activity. The *N*-terminal region links the two subdomains and crosses the catalytic site, forming favourable interactions with both the activation segment (Asp175 to Glu201, (Figure 1)) and a basic cluster of residues in the αC helix that recognise its substrates (Lys74 to Lys83, (Figure 2)), stabilising the active conformation; the close contacts of the *N*-terminal region and activation segment are unique to CK2 and may contribute to the constitutive activity of the protein [51].

**Figure 3:**
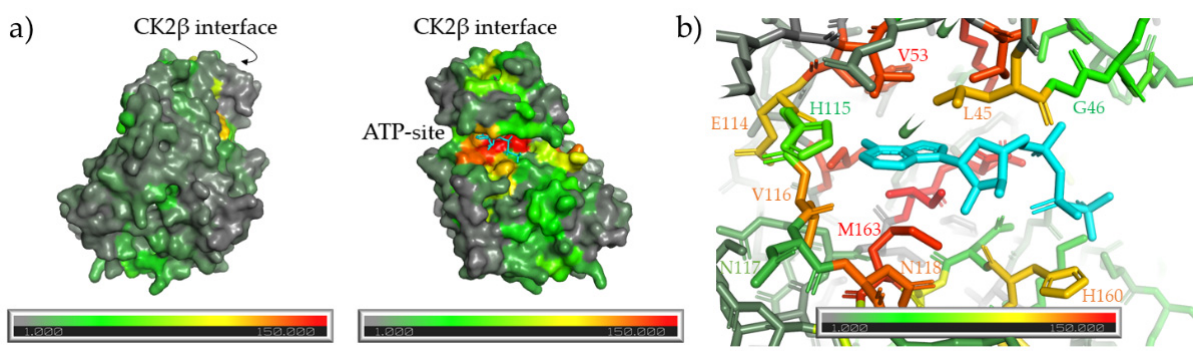
Heat map showing where known ligands bind to CK2α. 180 crystal structures of CK2α with ligands (excluding nucleotides binding to the ATP site) were analysed and interactions of the ligands with the kinase mapped. (**a**) Surface of CK2α (PDB: 5CU6) is coloured based on the number of ligands that are found within 4 Å of any atom in the underlying residue. Darkest red shows residues that contact ligands in 150 structures while grey residues make no direct ligand interactions, with green and yellow representing intermediate values. In (**b**) ADP molecule is shown as light blue sticks for reference. Residues with atoms which are frequently found within 4 Å of a ligand are shown in green, yellow, orange and red reflecting the number of structures in which this proximity was identified; key binding residues also frequently identified as being in close proximity to inhibitors ligands have been labelled.

The structure of the ATP site has led to it historically being the main site for which inhibitors were developed [52,53]. The heat maps in Figure 3 quantitatively highlight that the vast majority of CK2 inhibitors developed to date bind to the ATP-site of CK2α, a handful to areas surrounding the ATP-site and almost none to the opposite face of CK2α (Figure 3a). The constitutive nature of the protein means that the structure is always ready to accept an ATP-like molecule [54]. Additionally, the knowledge of the structure of both the active site and its natural partners, ATP or GTP, removes the problems associated with de novo inhibitor design [55,56]. As such, many ATP-competitive inhibitors of CK2 have been identified to date, including the orally available small molecule CX-4945 (Figure 2) which is currently in phase II clinical trials for the treatment of cholangiocarcinoma [45,57].

This review focuses on the most influential ATP-competitive inhibitors developed for the protein kinase CK2, their syntheses, advantages and disadvantages and the new approaches being utilised to render ATP-competitive inhibitors more successful. To the best of our knowledge, this is the only review that covers the discoveries in the field that have taken place in the last eight years [58].

## 2. History of ATP-Competitive Inhibitors of CK2

Over the past two decades, a vast amount of work has gone into developing potent and cell permeable ATP-competitive inhibitors for CK2. The most influential inhibitors previously developed can broadly be broken down into four main classes:

1. Halogenated compounds such as DRB and its derivatives [59,60]
2. Condensed polyphenolic compounds such as emodin and its derivatives [61,62]
3. Pyrazolo-triazines and pyrazolo-pyrimidines [63,64]
4. Indoloquinazolines such as CX-4945 [65]

**Figure 4:**
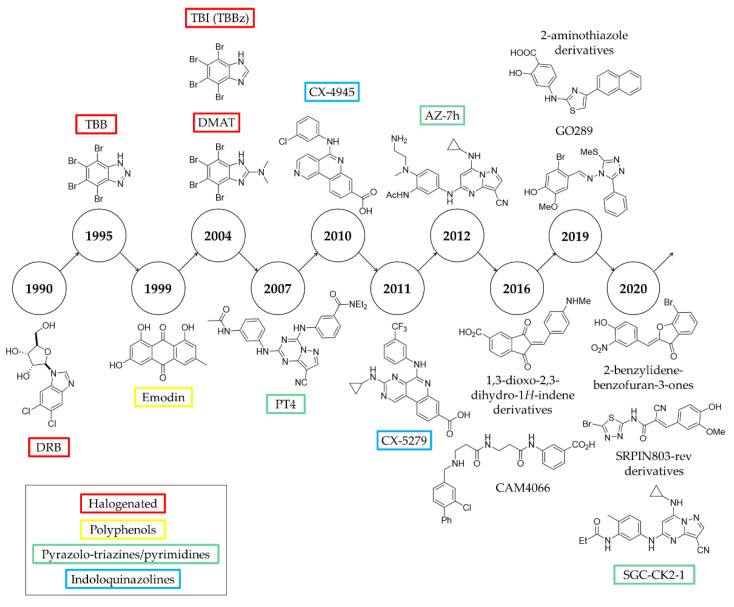
Timeline detailing the chronological development of the most influential CK2 ATP-competitive inhibitors and the most recent developments in the field. Halogenated compounds derived from DRB are outlined in red; polyphenols are outlined in yellow; pyrazolo-triazines and pyrazolo-pyrimidines are outlines in green; indoloquinazolines are outlined in blue.

The chronology of the most significant inhibitors developed thus far is detailed in Figure 4.

### 2.1. DRB

**Scheme 1:**
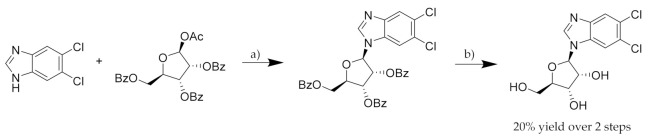
A synthetic route to 5,6-dichloro-1-β-D-ribofuranosylbenzimidazole (DRB). (**a**) *N*,*O*-bis-(trimethylsilyl)-acetamide, trimethylsilyl trifluoromethanesulfonate, MeCN, 130 °C, 5 min, microwave irradiation; (**b**) NH_3_, MeOH, 50 ℃, 16 h [67].

5,6-dichloro-1-β-D-ribofuranosylbenzimidazole (DRB) is a nucleoside analogue, first identified as being biologically active in 1954 [66]. The synthesis of DRB is well known in the literature with multiple facile routes reported, one of which is shown in Scheme 1 [67].

The inhibition of CK2 by DRB was detailed by Zandomeni et al. in 1986 [59]. However, DRB displays only modest activity against CK2 (IC_50_ = 15 μM) [59] and is capable of inhibiting multiple other kinases, such as CK1, with comparable efficacy [68]. In addition to this, DRB was also found to bind to the CK2α/β interface [69]. As such, due to its promiscuity both in binding to CK2 and other kinases, DRB has limited use today. Nevertheless, since its discovery, DRB has been the starting point of many studies to develop more potent and selective CK2 inhibitors, and many of the most widely used CK2 ATP-site inhibitors used currently are derived from DRB.

### 2.2. TBB

**Scheme 2:**
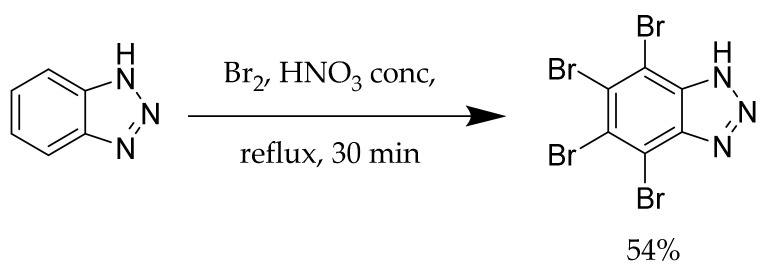
Synthesis of 4,5,6,7-tetrabromo-1H-benzotriazole (TBB) [71].

The first major advance on DRB came from two primary improvements being made: the removal of the sugar moiety and the replacement of the chlorine atoms with bulkier bromines leading to the development of 4,5,6,7-tetrabromo-1H-benzotriazole (TBB) [70]. The synthesis of TBB was first described by Wiley and Hussung in 1957 by the combination of bromine and nitric acid with benzotriazole (Scheme 2) [71].

Despite its somewhat simplified structure, TBB has a greatly improved potency compared to DRB (K*_i_* = 0.40 vs. 4.50 μM, respectively) [60]. It was originally believed that TBB was selective for CK2, as deemed against a panel of 30 kinases [72]. However, since then, the kinase panel available for testing has drastically increased [73] and in a subsequent screen against a panel of 70 protein kinases, seven were inhibited significantly (>90%, 10 μM of TBB) and six of these were inhibited more strongly than CK2 itself, which had 6% residual activity [74]. Another concern with TBB is the possibility of cumulative toxicity arising from its large number of halogen atoms although, thus far, this issue has not been seen and TBB was not found to be toxic to mice [52,75]. Despite its shortcomings, TBB was a drastic improvement on DRB and has been widely used in vitro and in vivo to gain a greater understanding of the biological role of CK2 [76,77,78].

### 2.3. TBI (TBBz) and DMAT

**Scheme 3:**
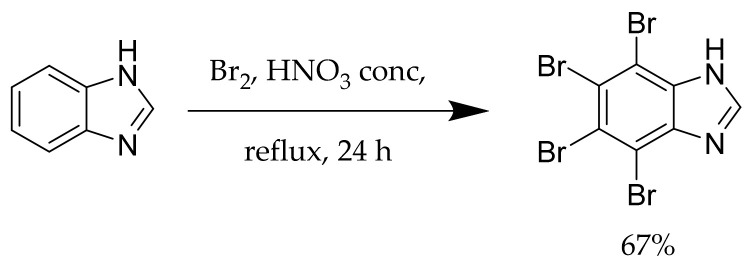
Synthesis of 4,5,6,7-tetrabromobenzimidazole (TBI) [79].

TBI (4,5,6,7-tetrabromobenzimidazole, also known as TBBz) is synthesised under the same conditions as TBB (Scheme 3) [79] and was first discovered as a CK2 inhibitor by Szyszka et al. in 1995 [70]. It was later revisited as a possible CK2 inhibitor simultaneously by Andrzejewska et al. and Zień et al. in 2003 [79,80]. TBI was found to be more active than the previous best inhibitor TBB (Table 1), as well as more selective, in particular against PK60S [79,80].

**Scheme 4:**
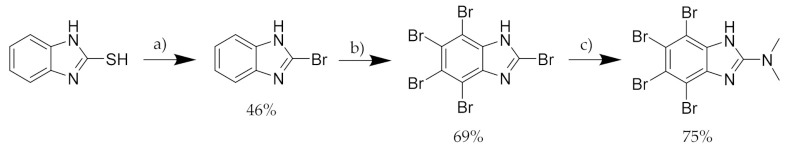
Synthesis of DMAT. (**a**) HBr, Br_2_, 1 h, 5–10 °C; (**b**) Br_2_, H_2_O, 24 h; (**c**) 30% ethanolic NHMe_2_, 110–115 ℃ [80,81].

A subsequent systematic structure-activity relationship study conducted on TBI and its derivatives by Pagano et al. led to the development of 2-dimethylamino-4,5,6,7-tetrabromo-1H-benzimidazole (DMAT) [81], which is significantly more potent than TBB (Table 1), particularly in cells [82,83]. DMAT can be synthesized in three steps from 2-mercaptobenzimidazole via the complete bromination of the imidazole followed by installation of the dimethylamine functionality (Scheme 4) [80,81].

Due to its significantly improved activity compared to TBB (Table 1), DMAT became the main ATP-competitive CK2 inhibitor used after its development and analysis in the early 2000s [84,85,86,87]. However, DMAT and TBI have both been shown to strongly inhibit PIM1, PIM2, PIM3, PKD1, HIPK2 and DYRK1a (all 10% or less residual activity with 10 μM of inhibitor), as well as cyclin A/CDK2 (~20% residual activity with 10 μM of inhibitor). TBB is more selective toward CK2, although it too has been found to inhibit PIM1 and PIM3 (<10% residual activity with 10 μM and 1 μM of inhibitor) [74]. Both DMAT and TBB have been widely used as inhibitors of CK2, although it is clear that a compromise between activity and selectivity must be made when using either of these compounds for biological analysis.

### 2.4. Polyphenols

**Figure 5:**
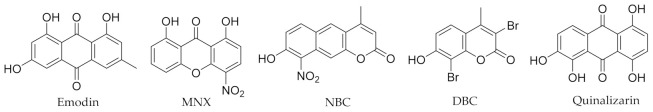
Structures of polyphenolic ATP-competitive inhibitors of CK2: emodin, MNX, NBC, DBC and quinalizarin [62,88,93].

Polyphenolic compounds, such as emodin (Figure 5), are common natural products that can be isolated from a variety of plants. The first polyphenol to be identified as a CK2 inhibitor was emodin which, up until this point, was known as an inhibitor of protein tyrosine kinases with IC_50′_s of 10–20 μM [62,88,89]. However, it was discovered in 1999 that emodin was more active against CK2 (IC_50_ = 2 μM, K*_i_* = 7.2 μM) [61]. It should be noted here that some studies have classed polyphenols and quinone derivatives as pan-assay interference compounds (PAINs) [90,91]. However, typically entire classes of compounds do not exhibit this behaviour, rather a select few [92]. Nevertheless, this may begin to explain the somewhat promiscuous activity of emodin.

A range of studies have aimed to improve the polyphenolic inhibitors of CK2, most notably identifying compounds 1,8-dihydroxy-4-nitroxanthen-9-one (MNX), 8-hydroxy-4-methyl-9-nitrobenzo[g]chromen-2-one (NBC), and 3,8-dibromo-7-hydroxy-4-methylchromen-2-one (DBC) (Figure 5) [62].

All three compounds exhibit improved activity compared to emodin as summarised in Table 2. DBC is the most potent analogue, however, it is poorly selective. DBC was found to significantly inhibit DYRK1a, MAPKAP-K1a, MSK1, and PRAK whereas MNX and NBC were much more selective; MNX only significantly inhibited one other enzyme, DYRK1a by 49%, and NBC did not inhibit any other proteins by more than 40% [62]. However, this study only screened the compounds against a panel of around 30 kinases and it is currently not known if they are equally selective against the rest of the kinome. MNX and NBC were tested in cells and shown to be equally effective at inducing cell death as TBB (DC_50_ = 17, 18 and 17 μM, respectively, Jurkat cells) [62].

Another polyphenolic compound structurally related to emodin is quinalizarin (1,2,5,8-tetrahydroxy-anthraquinone), identified as a CK2 ATP-site inhibitor through a computational screen [93]. Although structurally very similar to emodin, the activity of quinalizarin is significantly greater than that of its predecessor, as shown in Table 2. In addition to its high activity, quinalizarin also displays good selectivity; when tested against a panel of 72 kinases, quinalizarin only strongly inhibited CK2 (7% residual activity, 1 μM of quinalizarin), with no other kinase inhibited over 50% [93]. Finally, quinalizarin was shown to successfully induce apoptosis in HEK293T cells and was faster acting than TBB [93]. Thus, it represents the most promising candidate to date from the polyphenolic class of CK2 ATP-competitive inhibitors. Nevertheless, its activity against a larger proportion of the kinome still needs to be evaluated.

### 2.5. Pyrazolo-Triazines and Pyrazolo-Pyrimidines

**Scheme 5:**
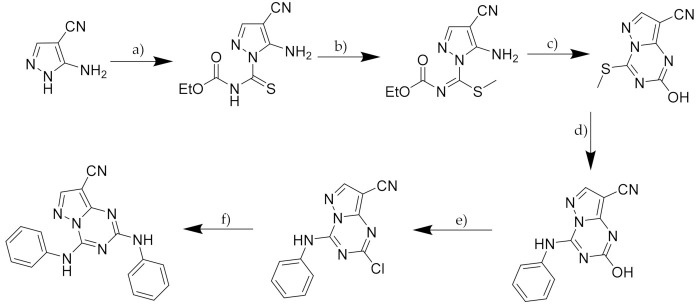
Synthetic route to the pyrazolo-triazine ATP-competitive CK2 inhibitor PT1. (**a**) (CO_2_Et)NCS, EtOAc/benzene, rt; (**b**) MeI, NEt_3_, THF; (**c**) DBU, DMF; (**d**) Aniline, 90 °C; (**e**) POCl_3_, reflux; (**f**) Aniline, ^i^Pr_2_NEt, NMP, 50 °C; no yields available as compounds were synthesised in 96-deep well plates and used without purification [63].

A new series of pyrazolo-triazines were designed as CK2 inhibitors by Nie et al. in 2007 [63]. A co-crystal structure of CK2 with a pyrazolo-triazine, herein referred to as PT1 (synthesis outlined in Scheme 5), indicated that the adenine binding site of CK2 was occupied by the pyrazolo-triazine core and the inhibitor was secured in the ATP-binding site by two hydrogen bonds in the hinge region of the protein [63].

Structure-activity relationship studies on PT1 led to the development of three lead compounds, differing in the substituent on one of the phenyl rings, namely methyl piperidine (PT2), a pyrrolidinyl amide (PT3) and *N,N-*diethylamide (PT4). All three compounds had nanomolar K_i_’s for CK2 but were only micromolar inhibitors of cell growth in both HCT116 and PC3 cell lines when tested in an MTT assay (Table 3) indicating that the physicochemical properties of the series required improvement to diminish this discrepancy [63].

Subsequent optimisation of the series led to the development of a second generation of pyrazolo-triazine inhibitors, including PT5 (Table 3) [94]. The planarity of the original compounds was decreased by macrocylisation with an alkyl side chain. This reduction in flatness appeared to successfully aid the compound’s cellular permeability and, despite a 10-fold reduced K*_i_* compared to the previously developed compounds, PT5 displayed comparable growth inhibition of cells (Table 3). Thus, the drop-off in activity between enzymatic and cellular assays was reduced [63,94]. Optimisation of the second generation’s binding affinity may lead to the development of potent and cell permeable CK2 inhibitors. However, to date, no such optimisation of constrained pyrazolo-triazines has been published. Additionally, no data are currently available on the selectivity profiles of the compounds.

**Figure 6:**
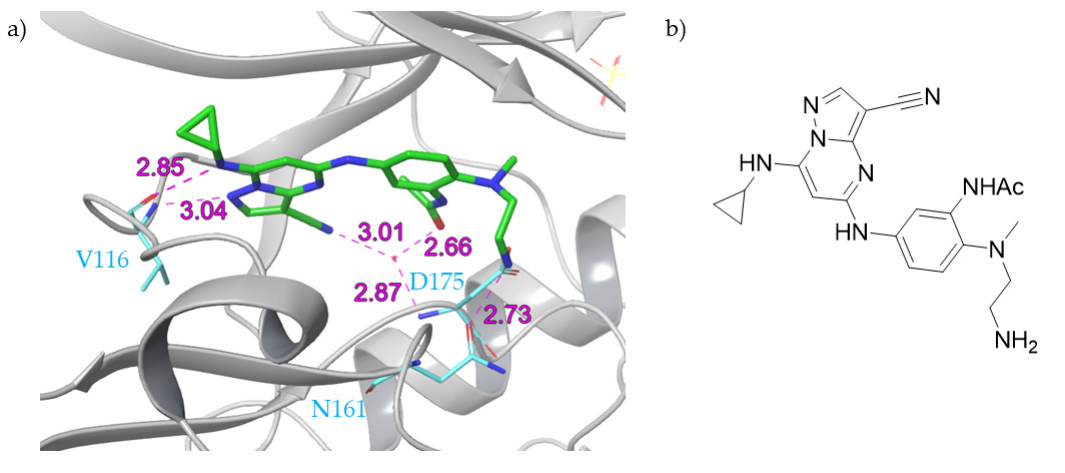
Depictions of the binding mode of AZ-7h in the ATP-site of CK2α and the chemical structure of AZ-7h. (**a**) Crystal structure of AZ-7h bound in the ATP active site of CK2α (PDB: 5H8E). Residues which form hydrogen bonds to the inhibitor are shown in cyan and the lengths of the hydrogen bonds given in Å. (**b**) Chemical structure of the pyrazolo-pyrimidine CK2 inhibitor AZ-7h.

A similar class of compounds, pyrazolo-pyrimidines, were identified by Dowling et al. using a kinase-focused subset screening approach followed by structure-activity relationship studies and crystallography to guide further development [64]. This optimisation ultimately led to the identification of a lead candidate, 7h (herein referred to as AZ-7h) (Figure 6), which has picomolar binding to CK2 (K_d_ = 6.33 pM) and nanomolar activity in cells (GI_50_ = 10 nM, HCT116 cells) [64,95]. Unfortunately, data for direct comparison with the pyrazolo-triazine series are not available. However, the compound’s selectivity was tested against a panel of 402 kinases and AZ-7h showed modest selectivity, with 12 kinases being inhibited by >50%, all of which were members of the CMGC family. Although displaying limited off-target activity, AZ-7h was active in the 10–20 nM range against Dapk2 and Dapk3 [95]. In vivo mouse studies showed poor oral bioavailability of AZ-7h, but therapeutic compound concentrations were achieved with intravenous and intraperitoneal injections, and the compound exhibited dose-dependent tumour growth inhibition [95]. The promising results of AZ-7h suggest that further optimisation of the pyrazolo-pyrimidine inhibitors should be strongly considered, with a focus on decreasing the activity against Dapk2 and Dapk3.

### 2.6. CX-4945

**Scheme 6:**
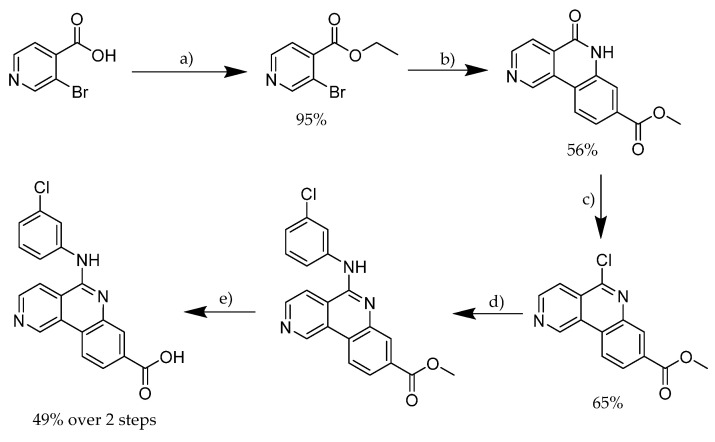
Synthetic route to CX-4945 as developed by Cylene Pharmaceuticals Inc. (**a**) EtOH, H_2_SO_4_ conc, reflux, 12 h; (**b**) 2-amino-4-methoxycarbonyl-phenylboronic acid, sodium acetate, 1,1′-bis(diphenylphosphino)ferrocene palladium (II) chloride, DCM, 125 °C, 12 h; (**c**) POCl_3_, reflux, 2 h; (**d**) meta-chloroaniline, *N*-methyl pyrrolidinone, 80 °C, 2 h; (**e**) NaOH, MeOH, 50 °C, 30 min [96].

CX-4945 (also known as Silmitasertib, ClinicalTrials.gov identifier: NCT02128282 (accessed on 27 June 2017)) was developed as an orally bioavailable CK2 inhibitor in 2010 by Siddiqui-Jain et al. [50]. Its synthesis was detailed in a patent by Cylene Pharmaceuticals Inc and is a 5-step route starting from 3-bromoisonicotinic acid (Scheme 6) [96].

CX-4945 is a potent CK2 inhibitor (IC_50_ = 1 nM, K*_i_* = 0.38 nM) [50] and it is postulated that its strong binding affinity arises from the formation of an ionic bridge with Lys68, in addition to hydrophobic interactions and hydrogen bonding with the hinge region of the active site [97]. The selectivity of CX-4945 was tested against a panel of 238 protein kinases, at a concentration 500-times greater than its IC_50_ against CK2, and seven kinases were found to be inhibited by over 90% [50]. However, despite the observed selectivity at relatively low concentrations, CX-4945 has actually been found to inhibit 12 other kinases with nanomolar activity, including CLK2 which is inhibited more strongly than CK2α under the same conditions [97,98,99,100,101,102]. Due to its high potency and favourable physicochemical properties, CX-4945 has been used extensively as a CK2 inhibitor, despite still not displaying the desired selectivity [46,103,104,105,106]. Unlike AZ-7h, CX-4945 was found to be orally bioavailable (ranging from 20–51% across species), representing a great advance in the field [97].

CX-4945 has also been seen to supress DNA damage repair-related elements when complexed with cisplatin-like constructs as a prodrug. The CX-4945-Pt(IV) prodrugs, CX-4945-cisplatin-Cl and CX-4945-DN604-Cl, had superior cytotoxicity to both cisplatin and the improved cisplatin-analogue DN604 and they were able to reverse drug resistance [107]. As a result of this promising behaviour, CX-4945 is currently in phase II clinical trials for the treatment of Cholangiocarcinoma in combination with cisplatin and gemcitabine [45,57].

**Figure 7:**
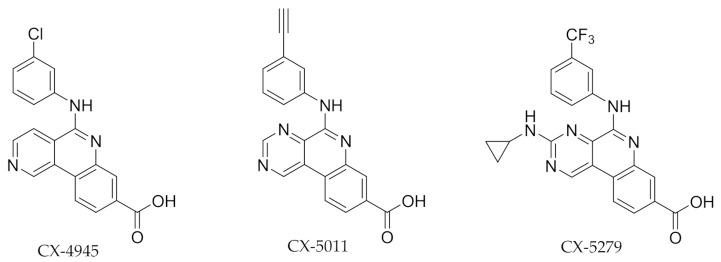
Structures of the improved ATP-competitive inhibitors CX-5011 and CX-5279 compared to CX-4945 [108].

A structure-activity relationship study on CX-4945 led to the development of CX-5011 and CX-5279 (Figure 7) which both contain a pyrimidine ring in place of the pyridine of CX-4945 [108]. Most promisingly, both compounds show a much greater specificity than CX-4945, with Gini coefficients of 0.735, 0.755 and 0.615 for CX-5011, CX-5279 and CX-4945, respectively (panel of 102 kinases); the improved Gini coefficients represent the lower activity of the compounds against other protein kinases. For example, the strong inhibition of CX-4945 for PIM1 (IC_50_ = 216 nM) is less marked for the two follow-up compounds (PIM1 IC_50_ = 2.5 μM and 8.5 μM for CX-5011 and CX-5279, respectively) [109].

Despite these more selective derivatives being identified, CX-4945 is still often seen as the gold standard of CK2 inhibitors due to its high activity and promising preliminary results in clinical trials. Nevertheless, its suboptimal selectivity is undesirable when a chemical probe for CK2 is needed.

## 3. Recent Developments

**Figure 8:**
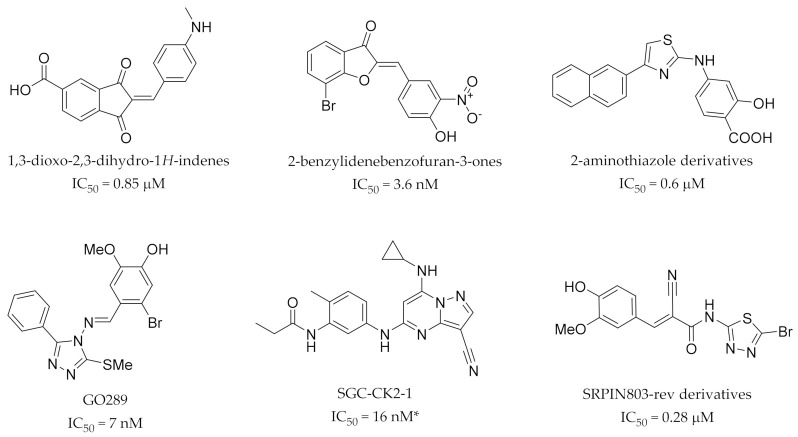
Structures of the most recently developed ATP-competitive CK2 inhibitors: 1,3-dioxo-2,3-dihydro-1H-indenes, 2-benzylidenebenzofuran-3-ones, 2-aminothiazoles, GO289, SGC-CK2-1 and SRPIN803-rev derivatives. *IC50 for SGC-CK2-1 is for CK2α’ [110,111,112,115,116,117].

Due to the promiscuity associated with the aforementioned inhibitors, there are a number of efforts ongoing to identify new series of CK2 ATP-competitive inhibitors. Some, such as a new series of 1,3-dioxo-2,3-dihydro-1*H*-indenes (Figure 8), represent novel scaffolds with moderate affinity for CK2 (micromolar IC_50′_s) and present the opportunity for optimisation to develop more potent and selective derivatives [110]. Others report nanomolar IC_50′_s in enzymatic assays, such as the 2-benzylidenebenzofuran-3-ones series of nanomolar inhibitors (Figure 8), but are yet to provide information on their selectivity, although this work is ongoing [111]. A series of 2-aminothiazole derivatives (Figure 8) with submicromolar potency were originally thought to be allosteric inhibitors of CK2 but were recently shown by Brear et al. to actually bind in the ATP active site [112,113,114]. Of all the recently identified inhibitors, three show the most promising results: GO289, SGC-CK2-1 and SRPIN803-rev derivatives (Figure 8) [115,116].

**Scheme 7:**
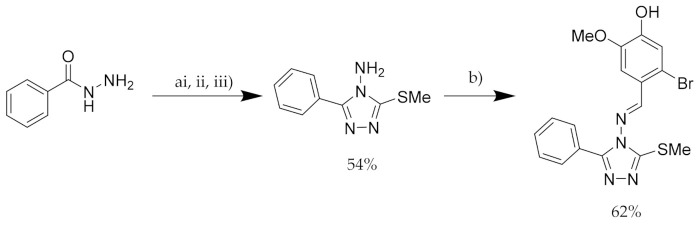
Synthesis of GO289. (**ai**) CS_2_, KOH, EtOH, 14 h; (**aii**) NH_2_NH_2_·H_2_O, reflux, 9 h; (**aiii**) MeI, KOH, H_2_O, 14 h; (**b**) 2-bromo-4-hydroxy-5-methoxybenzaldehyde, AcOH, 100 °C, 10 h [115].

Oshima et al. identified GO289 in 2019 through affinity-based target deconvolution. It is synthesised in four steps (Scheme 7); three steps to install the triazole ring flowed by condensation of the hydrazine and a benzylic aldehyde [115]. A structure-activity relationship study on GO289 found that the bromoguaiacol group is essential for activity whereas the para position of the substituted phenyl ring is modifiable [115].

**Figure 9:**
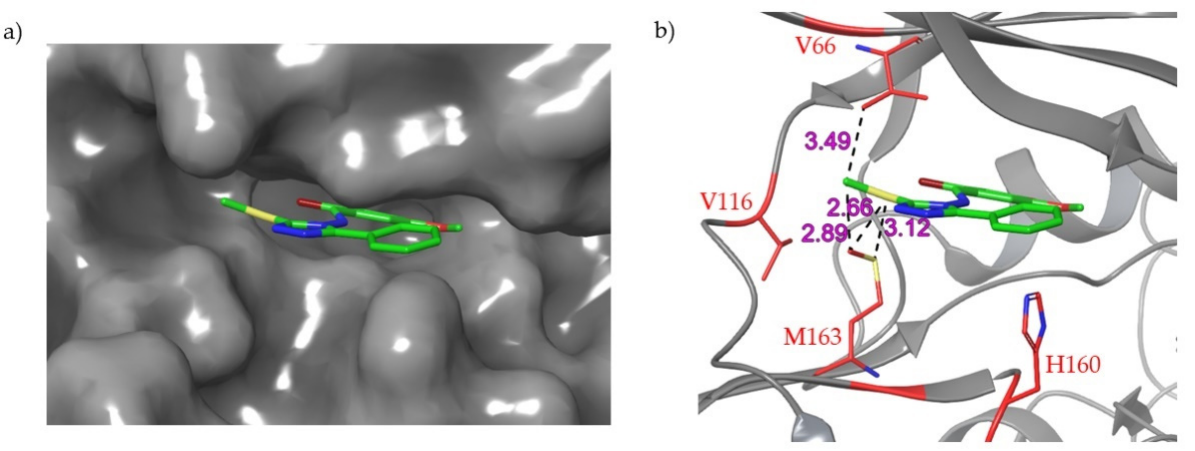
Binding mode of GO289 with CK2α and key interactions between the inhibitor and the ATP-site. (**a**) Structure of GO289 bound in the ATP-site of CK2α (PDB: 6A1C). (**b**) Interactions between CK2-specific residues and GO289. The key interacting residues are shown in red, namely H160, M163, V116 and V66. Hydrogen bond lengths are given in Å.

GO289 was shown to have very good activity (IC_50_ = 7 nM) as well as high selectivity for CK2; the second most inhibited protein, PIM2, is much more weakly inhibited by GO289 than CK2 (PIM2 IC_50_ = 13 μM). Most interestingly, the X-ray crystal structure of GO289 in complex with CK2α indicated key interactions between the compound and residues which are specific to CK2 (V66, V116, H160, M163), and no interactions with the highly conserved hinge region (Figure 9) [115]. Most CK2 ATP-competitive inhibitors, such as CX-4945, engage with the hinge region of the protein and these interactions can compensate for the loss of hydrophobic contacts between inhibitors and other proteins with a V66A mutation (e.g., DYRK, HIPK, PIM and CLK family kinases) [109,118].

**Scheme 8:**
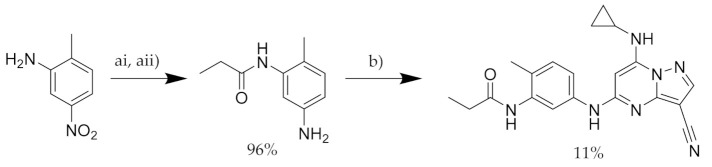
Synthesis of SGC-CK2-1. (**ai**) Propinoyl chloride, NEt_3_; (**ii**) H_2_, 10% Pd/C, MeOH; (**b**) 5-chloro-7-(cyclopropylamino)pyrazolo[1,5-*a*]pyrimidine-3-carbonitrile, KF, NMP, 140 °C [117].

In 2020, Wells et al. published a structure-activity relationship study on a series of pyrazolo-pyrimidines based upon AZ-7h, which was discussed earlier [64,95,117]. The lead compound developed, SGC-CK2-1 (previously compound 24), was synthesised via acylation of the aniline followed by reduction of the nitro group and coupling with the pyrazolo-pyrimidine core (Scheme 8) [117]. SGC-CK2-1 showed strong inhibition of both CK2 catalytic subunits in an intracellular nanoBRET assay on HEK-293 cells; IC_50_ = 36 nM and 16 nM for CK2α and CK2α’, respectively while CX-4945, for comparison, had an IC_50_ of 45 nM (CK2α’) in this assay. Promisingly, SGC-CK2-1 exhibited much higher selectivity for CK2 than CX-4945 when tested against a panel of 403 kinases; SGC-CK2-1 inhibited three kinases > 90% at 1 μM (2 of which were CK2α and CK2α’) while CX-4945 inhibited 28 kinases under the same conditions [117]. Curiously, when tested in both HCT-116 and U-87 MG cells in-house, SGC-CK2-1 showed no antiproliferative activity (as did also CX-4945, unexpectedly) while showing 1.9 μM and 10 μM IC_50′_s for the same cell lines in a 140 cancer cell line panel. Additionally, no caspase 3/7 activation was observed with SGC-CK2-1 (tested up to 10 μM). The authors suggest that these results imply that antiproliferative activity exhibited by less selective CK2 inhibitors arises due to inhibition of off-target kinases as opposed to CK2 inhibition. As CX-4945 also showed no antiproliferative activity in their cellular assays and the externally run cell panel assay results are contradictory to this, these results must be investigated further before reliable conclusions can be drawn [117].

**Scheme 9:**
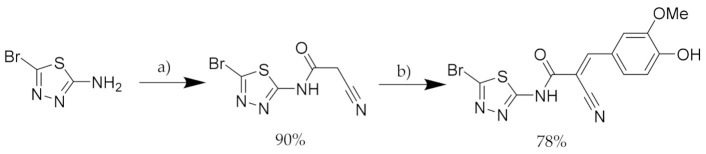
Synthesis of lead compound in the series of SRPIN803-rev derivatives. (**a**) 2-cyanoacetic acid, trifluoroacetic anhydride, THF, 1 h; (**b**) 4-hydroxy-3-methoxybenzaldehyde, AcOH, NH_4_OAc, MeCN, 90 °C, 3 h [116].

SRPIN803 was previously identified as a dual inhibitor of SRPK1 and CK2, although it showed 10-fold greater inhibition of CK2 (IC_50_ = 203 nM and 2.4 μM for CK2 and SRPK1, respectively) [119]. In 2020, the crystal structure of SRPIN803 in complex with CK2α revealed that the compound was in the open form (the thiadiazole nitrogen had not reacted with the nitrile carbon as reported previously) [119]. This led to the renaming of the open form of the compound as SRPIN803-rev for clarity. A series of derivatives were subsequently synthesised (synthetic route for lead compound is shown in Scheme 9) and found to have moderate inhibitory activity against CK2 (IC_50_ = 0.28 μM for lead compound) as well as good selectivity (only CK2α inhibited over 50% at 1 μM in a 320-kinase panel) and cellular activity (DC_50_ = 12.8 μM, Jurkat cells).

**Figure 10:**
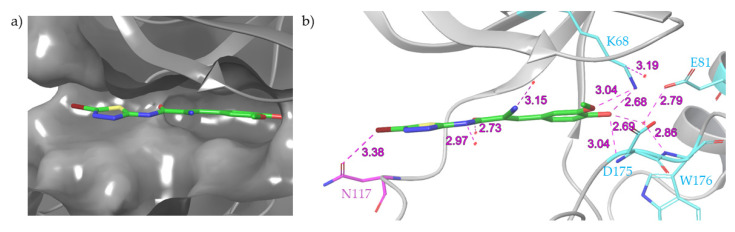
Binding mode and key interactions between the most promising SRPIN803-rev derivative and CK2α. (**a**) Crystal structure of the lead compound in the series of SRPIN803-rev compounds (compound 4 in the paper) bound to CK2α (PDB: 6RFE). (**b**) Ribbon depiction of structure highlighting the key binding interactions and the unusual binding mode of the compounds: the inhibitor protrudes out of the active site past the hinge region. Residues involved in hydrogen bonding with the ligand are shown in cyan; residues involved in halogen bonding are shown in magenta; bond distances are displayed in Å.

These compounds constitute the first CK2 inhibitors reported to protrude from the active site of the protein on the top of the hinge region (Figure 10), and it is this property which is believed to aid their selectivity [116]. CK2 has a flexible hinge/αD region which is capable of assuming a large number of conformations, the most common two in apo structures being: a closed form, which is seen in the majority of protein kinases [17], and an open form which is very rare [120]. The authors claim that the SRPIN803-rev derivatives bind to the open conformation of the hinge/αD region and that it is this binding mode which provides the superior selectivity of the series compared to the majority of CK2 ATP-competitive inhibitors [116].

GO289, SGC-CK2-1 and the SRPIN803-rev derivatives are the most active and selective newly identified CK2 ATP-competitive inhibitors with the most complete characterisation of their binding modes and selectivity. However, all the newly identified series and scaffolds represent an opportunity for the development of potent and selective chemical probes with further optimisation. It is interesting to note that half of the newly identified scaffolds contain α,β-unsaturated carbonyls (Figure 8) and as such may be capable of reacting with a variety of thiols in vivo; thorough investigation of this reactivity should be considered before further development of these series. Nevertheless, the number of newly developed series, as well as the recent success of GO289, SGC-CK2-1 and the SRPIN803-rev derivatives, give hope that a highly selective chemical probe for CK2 will be obtainable with an ATP-competitive inhibitor in the near future.

## 4. Dual Inhibitors

**Figure 11:**
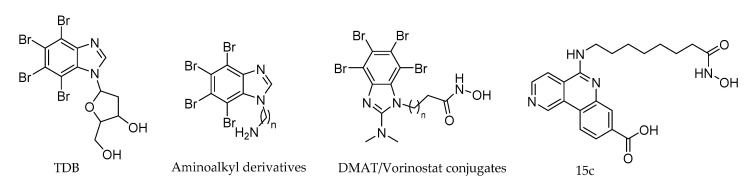
Structures of dual CK2 inhibitors. TDB and the aminoalkyl derivatives target CK2 and PIM1. DMAT/Vorinostat conjugates and 15c target CK2 and HDAC [121,123,125,126].

An alternative approach to the problems of ATP-competitive CK2 inhibitors was taken by Cozza et al. whereby they utilised the promiscuity of TBI towards other enzymes, specifically PIM1. The promiscuous activity of TBI was curtailed by functionalisation of the benzimidazole core with a deoxyribose moiety to form 1-(β-D-2′-deoxyribofuranosyl)-4,5,6,7-tetrabromo-1H-benzimidazole (TDB) (Figure 11) [121]. Unlike TBI, TDB is mostly selective for CK2 and PIM1 (IC_50_ = 32 nM and 86 nM for CK2 and PIM1, respectively), although it does also inhibit CLK2 comparably (IC_50_= 20 nM) [121,122]. Cell testing showed that the pro-apoptotic activity of TDB was superior to “selective” inhibitors of either CK2 or PIM1, which the authors suggest is due to the synergistic effect of simultaneous inhibition of CK2 and PIM1. Despite CX-4945 having a greater in vitro potency against CK2 than TDB and similar activity against PIM1 (CK2 IC_50_ = 2.5 nM and 32 nM for CX-4945 and TDB, respectively; PIM1 IC_50_ = 216 nM and 86 nM for CX-4945 and TDB, respectively), TDB was found to be more cytotoxic than CX-4945 [122].

Chojnacki et al. also published research in 2018 detailing an analogous series of aminoalkyl CK2/PIM1 dual inhibitors based on the structure of TBI (Figure 11) [123]. However, these compounds do not have as favourable binding affinities for CK2α as those aforementioned; TDB K*_i_* = 15 nM, 40 nM (CK2α and PIM1, respectively) whereas for the lead aminoalkyl-substituted derivative, K*_i_* = 770 nM, 270 nM (CK2α and PIM1, respectively) [122,123].

Recently, a similar approach was taken by the Ramos group, this time combining CK2 and HDAC inhibition [124,125]. They combined the structure of vorinostat, a known selective HDAC inhibitor, with that of DMAT to create a series of dual CK2/HDAC inhibitors [124]. Optimisation led to the development of a series of compounds with low micromolar activity in enzymatic assays as well as low micromolar LC_50_ values for a variety of cell lines (Figure 11) [125]. In 2020, the same group published an alternative series of CK2/HDAC inhibitors in which the DMAT-derived portion of the compounds was replaced with the much more potent CK2 inhibitor CX-4945. The lead compound developed, 15c (Figure 11), exhibited significantly greater inhibitory activity against CK2 and HDAC1 than the reference compounds (3.0 and 3.5 times higher activity than CX-4945 and vorinostat, respectively) as well as micromolar activity in cell-based cytotoxicity assays [126].

The development of dual functional inhibitors is still in its infancy. Nevertheless, promising early results suggest that it may be a viable strategy to utilise the promiscuity of CK2 ATP-competitive inhibitors for maximum therapeutic effect. This approach may result in the development of highly effective anti-proliferative compounds with minimal off-target effects if the promiscuous activity of the inhibitors can be limited to the desired kinases; however, this strategy is not widely applicable for the development of chemical probes as the observed effects of the compounds will depend upon the combined functions of the kinases targeted.

## 5. Bi-Substrate Inhibitors

Another method for CK2 inhibition combines both ATP- and substrate-competitive inhibitors to give bi-substrate inhibitors. Bi-substrate inhibitors are made up of two distinct moieties: typically, an ATP-site inhibiting fragment for protein kinases and a motif resembling a substrate of the enzyme [127]. These inhibitors are discussed in detail in a recent review of non-ATP-competitive CK2 inhibitors and readers are redirected here for a detailed discussion [128]. For interest, a brief overview will be provided.

**Figure 12:**
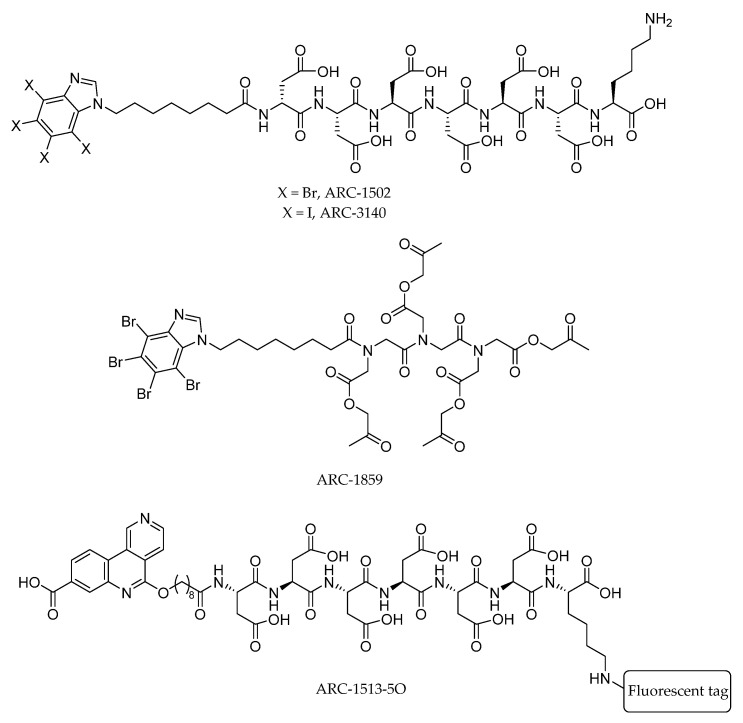
Structures of bi-substrate inhibitors of CK2: ARC-1502, ARC-1859, ARC-1513-5O and ARC-3140 [129,130,131,132].

The first bi-substrate inhibitor successfully developed was ARC-1502 (Figure 12), constituting the ATP-competitive inhibitor TBI bound to an acidic peptide [129]. ARC-1502, had a strong binding affinity for CK2α (K*_i_* = 0.5 nM) alongside good selectivity [129]. However, it is not proteolytically stable or cell permeable, limiting its utility [130]. Subsequently, a cell permeable and stable “pro-drug” was developed by use of a peptoid chain and masking of the aspartic acid side chains with acetoxymethyl esters to give ARC-1859 (Figure 12), which was shown to be active in cells [130].

In 2017, the bi-substrate inhibitor ARC-1513-5O (Figure 12) was developed which uses CX-4945 as the ATP-competitive inhibitor. Despite showing picomolar binding to CK2, ARC-1513-5O showed only moderate selectivity, highlighting the need to use a selective ATP-site inhibiting portion of the molecule to gain a selective bi-substrate inhibitor [131].

Pietsch et al. recently developed ARC-3140 (Figure 12) which utilises tetraiodobenzimidazole as the ATP-site inhibitor. ARC-3140 has picomolar binding to CK2 but was shown to also bind at two other locations on CK2α [132].

The handful of bi-substrate inhibitors developed thus far are proof that the strategy is possible. However, the compounds need further development and analysis before they can be truly effective chemical probes. In particular, the selectivity profiles of the compounds need to be evaluated in detail. It is probable that the selectivity of the molecule will be determined by the most potent portion of the molecule. Therefore, for this strategy to produce highly selective compounds, the selectivity of ATP-competitive inhibitors must be enhanced, or the affinity of substrate-competitive inhibitors must be increased. Additionally, none of the crystal structures reported to date have well defined density arising from the substrate binding site portion of the molecule [132]. Overcoming this issue and obtaining a well-defined crystal structure for such compounds would aid future development and facilitate structure-guided design.

## 6. Inhibitors Extending Outside of the ATP-Site

**Figure 13:**
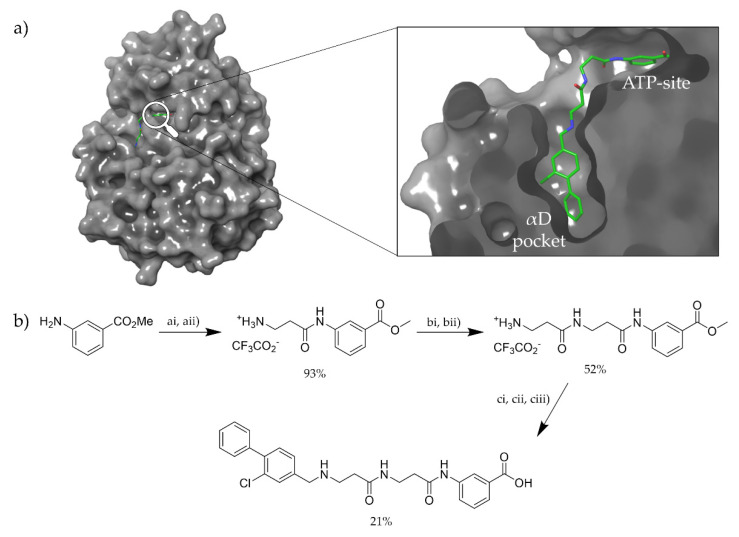
(**a**) Structure of CAM4066 bound to CK2α, spanning the αD pocket and the ATP-site (PDB: 5CU3). (**b**) Synthesis of CAM4066; (ai) boc-β-Ala, DCC, DMAP, CH2Cl2, 16 h; (aii) TFA/CH2Cl2, 30 min; (bi) boc-β-Ala, HATU, DMAP, DMF, 16 h; (bii) TFA/CH2Cl2, 30 min; (ci) 2-chloro-[1,1′-biphenyl]-4-carbaldehyde, Na(AcO)3BH, Et3N, MeOH, 18 h; (cii) LiOH, H_2_O/THF, 2 h; (ciii) 4 M HCl in dioxane, 10 min [102].

An allosteric inhibitor developed by the Hyvönen and Spring groups was found to bind in the recently discovered αD pocket of CK2 with a K_d_ of 270 μM but no inhibition of CK2 was observed [102]. Due to the proximity of the αD pocket to the ATP-site, the compound was linked to a fragment that bound weakly to the ATP-site with the rationale that selectivity for CK2 could be achieved as the majority of the binding affinity of the compound would arise from the allosteric portion of the molecule which binds in the poorly conserved αD pocket. The resulting compound, CAM4066, synthesized as outlined in Figure 13, showed subnanomolar activity in enzymatic assays and low micromolar cellular activity (K_d_ = 320 nM, IC_50_ = 370 nM, and a GI_50_ of 8.8 μM (for the methyl-ester prodrug)) [102,133]. Most promisingly, CAM4066 was shown to be selective with a Gini coefficient of 0.82 (52 kinase panel, 2 μM CAM4066) [102]. The high selectivity combined with the promising biological results of CAM4066 suggests that the unique binding mode of the molecule (Figure 13) is an effective strategy to obtain both strong inhibition of activity and selectivity amongst the kinome when developing protein kinase inhibitors and, thus, it may represent a promising design strategy to be applied to other kinases.

## 7. Conclusions

Despite their diverse and numerous roles in biology, protein kinases are a tight-knit family of enzymes. This poses numerous challenges for scientists trying to modulate their activity to decipher their specific roles and interactions, and for pharmaceutical development. ATP-competitive inhibitors are one of the most explored set of compounds via which to modulate their activities yet, due to the highly conserved nature of the ATP-active site amongst the kinome, they are often plagued by poor selectivity and off-target effects. This is true for the protein kinase CK2, for which a truly selective, highly potent and cell permeable ATP-competitive inhibitor is yet to be developed. However, continued research in the field, optimisation of existing inhibitors and the identification of novel scaffolds and series of compounds is providing hope that a good chemical probe will be obtained in the near future. Additionally, three alternative strategies are being used to try and circumvent the issues typically observed with ATP-competitive inhibitors: dual functional inhibitors, bi-substrate inhibitors and the combination of allosteric and ATP-site inhibitors. Although the fields are in their infancy, all three show encouraging initial results and, as such, may represent promising strategies to be applied to the modulation of a variety of other protein kinases.

In this review, we have described the challenges associated with developing ATP-competitive protein kinase inhibitors, utilising the kinase CK2 as an example; downfalls of historical CK2 inhibitors were highlighted, alongside an overview of the new discoveries in the field over the past eight years, including alternative strategies for inhibition utilising the ATP active site. Although a fully selective ATP-competitive probe for CK2 has yet to be identified, recent advances in the field put the end in sight, and it is hoped that the pitfalls and successes observed for CK2 inhibitors may provide inspiration for the development of effective modulators of other protein kinases for application in biochemistry, chemical biology and drug development.

## Figures and Tables

**Table 1:** Comparison of the structures and activities of CK2 ATP-competitive inhibitors DRB, TBB, TBI and DMAT [60,82].

Compound	Structure	CK2 K*_i_* (μM)
**DRB**	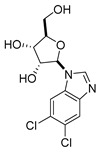	4.50
**TBB**	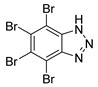	0.40
**TBI**	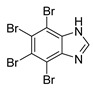	0.30
**DMAT**	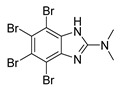	0.04

**Table 2:** Comparison of IC_50_ and K_i_ values for emodin and its improved derivatives [61,62,93]. Emodin was tested at 10 μM on a panel of 33 kinases; CK2 was only inhibited by 49% [60]. MNX, NBC and DBC were tested at 10 μM against a panel of 33 kinases [62]. Quinalizarin was tested at 1 μM on a panel of 75 kinases [93].

Compound	IC_50_ (μM)	K*_i_* (μM)	Number of other Proteins Inhibited > 40% (Concentration Tested)
**Emodin**	~2	7.2	2 (10 μM)
**MNX**	0.40	0.80	1 (10 μM)
**NBC**	0.30	0.22	0 (10 μM)
**DBC**	0.10	0.06	4 (10 μM)
**Quinalizarin**	0.11	0.055	1 * (1 μM)

* Quinalizarin was tested on a panel of 75 kinases; emodin, MNX, NBC and DBC were tested against 33 kinases.

**Table 3:** Comparison of the K_i_’s and IC_50′_s (HCT116 and PC3) of four pyrazolo-triazine CK2 inhibitors: PT2, PT3, PT4 and PT5 [63,94].

Compound	CK2 K*_i_* (nM)	HCT116 IC_50_ (μM)	PC3 IC_50_ (μM)
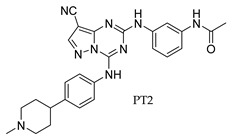	0.21	1.06	1.4
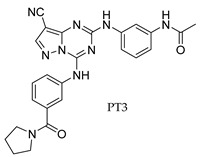	0.70	1.4	0.78
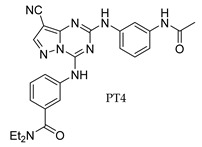	0.37	0.76	0.61
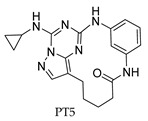	24	0.29	0.88

## Data Availability

Data sharing not applicable.
